# Supplementary material for: Thermoelectric active cooling for transient hot spots in microprocessors
Source: Nat Commun. 2024 May 20;15:4275. doi: 10.1038/s41467-024-48583-9 (PMC11106063; doi:10.1038/s41467-024-48583-9)
Supplement: Supplementary file 1 — Supplementary Information [file 41467_2024_48583_MOESM1_ESM.pdf]

Supplementary Information

Thermoelectric Active Cooling for Transient Hot Spots in Microprocessors

Yihan Liu[1], Hao-Yuan Cheng[2], Jonathan A. Malen[2, 3, *], Feng Xiong[1, *]

[1]Department of Electrical and Computer Engineering, University of Pittsburgh, Pittsburgh, PA 15261, USA.

[2]Department of Mechanical Engineering, Carnegie Mellon University, Pittsburgh, PA 15213, USA.

[3]Department of Materials Science and Engineering, Carnegie Mellon University, Pittsburgh, PA 15213, USA.

*Author to whom correspondence should be addressed. Emails: jonmalen@andrew.cmu.edu (J. A. Malen), f.xiong@pitt.edu (F. Xiong)

Supplementary Information Content:

1. **Derivation of Analytical Solution to the First Harmonic Temperature Response of Transient Active Cooling**
2. **Effect of DC Current on Transient Active Cooling Performance**
3. **Reduction of Thermal Boundary Conductance at Ni/p-Si Interface After Annealing**
4. **Measurement of T_δ's Phase and Phase Distortion Resulting from Device's Parasitic Inductance**
5. **Derivation of COP_{trans} at High Frequency**
6. **Comparison of COP_{trans} between p-Si and p-Bi_2Te_3 active cooling devices**
7. **Transient Temperature Cancellation at High Frequency (> 100 kHz)**
8. **Discussion on the Simplification of Neglecting Higher Order Transient Temperature Components in Model Study**

Supplementary Note 1: Derivation of Analytical Solution to the First Harmonic Temperature Response of Transient Active Cooling

The foundation of our analysis is rooted in the heat diffusion equation that governs a silicon layer, serving as a representation of the TED. This equation accounts for the effects of volumetric Joule heating and is articulated as follows:

$$\frac{1}{\alpha_{Si}}\frac{\partial T}{\partial t} = \nabla^2 T + J^2\rho_{Si}/\kappa_{Si} \qquad (S1)$$

where $T(r,z,t)$ is the temperature profile of the silicon layer, r and z represent the spatial coordinates as illustrated in Fig. 1b. The parameter α_{Si} denotes the thermal diffusivity of Si and t denotes time. The current density flowing through the layer is represented by J, while ρ_{Si} denotes the resistivity of silicon, and κ_{Si} represents the thermal conductivity of silicon. We decomposed the current and temperature profile into two components: a time steady-state component (0ω) and a first-order harmonic component (1ω) represented mathematically as $J = J_{0\omega} + J_{1\omega}\, sin(\omega t)$ and $T = T_{0\omega}(r,z) + T_{1\omega}(r,z) sin(\omega t)$, respectively. We then applied a Fourier transformation to Equation (S1) and obtained the following two heat equations for the 0ω and 1ω components.

$$0 = \nabla^2 T_{Si0\omega} + (J_{0\omega}^2 + J_{1\omega}^2/2)\rho_{Si}/\kappa_{Si} \qquad (S2)$$

$$\frac{i\omega}{\alpha_{Si}} T_{Si1\omega} = \nabla^2 T_{Si1\omega} + 2J_{0\omega}J_{1\omega}\rho_{Si}/\kappa_{Si} \qquad (S3)$$

Equations of heat diffusion in metal can be obtained from the same analysis but ignoring Joule heating since $\rho_m \approx 0$.

$$0 = \nabla^2 T_{m0\omega} \qquad (S4)$$

$$\frac{i\omega}{\alpha_m} T_{m1\omega} = \nabla^2 T_{m1\omega} \qquad (S5)$$

Table S1 lists the boundary condition equations for the top surface, interface between metal and Si, and bottom surface, where P is power of heat source, w_0 is the $1/\mathrm{e}^2$ spot radius, z_1 is the thickness of metal layer, z_2-z_1 is the thickness of Si layer, S is the Seebeck coefficient of Si, q''_{z_1} is the heat flux at silicon-metal interface, G is the silicon-metal interface thermal

conductance and T_c is the constant temperature, boundary condition at the bottom surface (T_c = 300 K).

**Top surface** heating by gaussian periodic source	(0ω)	$-\kappa_m \frac{\partial T_{m0\omega}}{\partial z} = \frac{2P_{0\omega}}{\pi w_0^2} \exp(-2r^2/w_0^2)$
	(1ω)	$-\kappa_m \frac{\partial T_{m1\omega}}{\partial z} = \frac{2P_{1\omega}}{\pi w_0^2} \exp(-2r^2/w_0^2)$
**Interface between metal and silicon** ($z = z_1$)	(0ω)	$-\kappa_m \frac{\partial T_{m0\omega}}{\partial z} = -\kappa_{Si} \frac{\partial T_{Si0\omega}}{\partial z} + SJ_{0\omega} T_{Si0\omega}$
	(1ω)	$-\kappa_m \frac{\partial T_{m1\omega}}{\partial z} = -\kappa_{Si} \frac{\partial T_{Si1\omega}}{\partial z} + S(J_{0\omega} T_{Si1\omega} + J_{1\omega} T_{Si0\omega})$
	(0ω)	$T_{m0\omega} = T_{Si0\omega} + q''_{z_1 0\omega}/G$
	(1ω)	$T_{m1\omega} = T_{Si1\omega} + q''_{z_1 1\omega}/G$
**Bottom surface** ($z = z_2$)	(0ω)	$T_{Si0\omega} = T_c$
	(1ω)	$T_{Si1\omega} = 0$

Table S1. Boundary condition equations for TED in time steady-state and the first harmonic state

Next, we applied the Hankel transform, as defined in Equation (S6) to Equations (S2)-(S5), to transform them from partial differential equations to ordinary differential equations.

$$\hat{T}(k,z) = \int_0^{+\infty} r \cdot B_0(kr) \cdot T(r,z) dr \tag{S6}$$

The hat symbol appearing above a variable indicates that it represents the Hankel transformation of that variable. B_0 represents the 0 order Bessel function of the first kind. The transformed heat equations are given by Equations (S7) and (S8), where $\delta(k)$ represents the Dirac delta function and $\delta(k)/k$ is the Hankel transformation of a constant.

$$0 = -k^2 \hat{T}_{Si0\omega} + \frac{\partial^2 \hat{T}_{Si0\omega}}{\partial z^2} + ((J_{0\omega}^2 + J_{1\omega}^2/2)\rho_{Si}/\kappa_{Si})\delta(k)/k \tag{S7}$$

$$\frac{i\omega}{\alpha_{Si}} \hat{T}_{Si1\omega} = -k^2 \hat{T}_{Si1\omega} + \frac{\partial^2 \hat{T}_{Si1\omega}}{\partial z^2} + (2J_{0\omega}J_{1\omega}\rho_{Si}/\kappa_{Si})\delta(k)/k \tag{S8}$$

The general solutions to these two ordinary differential equations are shown in Equations (S9) and (S10), where $\hat{T}^+$and $\hat{T}^-$are coefficients determined by the boundary conditions, and $u^2 = i\omega/\alpha_{Si} + k^2$.

$$\hat{T}_{Si0\omega} = \hat{T}_{Si0\omega}^+ \exp(ikz) + \hat{T}_{Si0\omega}^- \exp(-ikz) + ((J_{0\omega}^2 + J_{1\omega}^2/2)\rho_{Si}/\kappa_{Si})\delta(k)/k^3 \tag{S9}$$

$$\hat{T}_{Si1\omega} = \hat{T}_{Si1\omega}^+ \exp(iuz) + \hat{T}_{Si1\omega}^- \exp(-iuz) + (2J_{0\omega}J_{1\omega}\rho_{Si}/\kappa_{Si})\delta(k)/ku^2 \tag{S10}$$

These equations can be extended to the metal layer by substituting the relevant material parameters and setting $\rho = 0$. Hankel transformations of the boundary conditions yield $\hat{T}^+$and $\hat{T}^-$. The transformed boundary conditions are listed in Table S2:

**Top Surface**	(0ω)	$-\kappa_m \frac{\partial \hat{T}_{m0\omega}}{\partial z} = P_{0\omega} \frac{1}{2\pi} \exp\left(-\frac{1}{8} k^2 w_0^2\right)$
	(1ω)	$-\kappa_m \frac{\partial \hat{T}_{m1\omega}}{\partial z} = P_{1\omega} \frac{1}{2\pi} \exp\left(-\frac{1}{8} k^2 w_0^2\right)$
**Interface between metal and silicon**	(0ω)	$-\kappa_m \frac{\partial \hat{T}_{m0\omega}}{\partial z} = -\kappa_{Si} \frac{\partial \hat{T}_{Si0\omega}}{\partial z} + S J_{0\omega} \hat{T}_{Si0\omega}$
($z = z_1$)	(1ω)	$-\kappa_m \frac{\partial \hat{T}_{m1\omega}}{\partial z} = -\kappa_{Si} \frac{\partial \hat{T}_{Si1\omega}}{\partial z} + S\left(J_{0\omega} \hat{T}_{Si1\omega} + J_{1\omega} \hat{T}_{Si0\omega}\right)$
	(0ω)	$\hat{T}_{m0\omega} = \hat{T}_{Si0\omega} + \widehat{q''}_{z_1 0\omega}/G$
	(1ω)	$\hat{T}_{m1\omega} = \hat{T}_{Si1\omega} + \widehat{q''}_{z_1 1\omega}/G$
**Bottom Surface**	(0ω)	$\hat{T}_{Si0\omega} = \hat{T}_c\, \delta(k)/k$
($z = z_2$)	(1ω)	$\hat{T}_{Si1\omega} = 0$

Table S2. Boundary condition equations for TED in time steady-state and the first harmonic state after undergoing Fourier and Hankel transformations

Finally, the inverse Hankel transformation of $\hat{T}$ $(z = 0)$ is applied to determine the temperature profile at the top surface of the metal layer. This is given by,

$$\theta(r) = \int_0^{+\infty} k B_0(rk) \hat{T} dk = \lim_{k \to 0} k\, \hat{T} + \int_{0^+}^{+\infty} k B_0(rk) \hat{T} dk \quad \text{(S11)}$$

The first term on the right-hand side is the inverse Hankel transform of components of $\hat{T}$ that contain $\delta(k)$. This term represents the surface temperature profile when AC current is driven through the device without laser heating at the top surface. For the first harmonic temperature result the analytical solution of $\lim_{k \to 0} k\, \hat{T}_{1\omega} \delta(k)$ can be expressed as

$$\lim_{k\to 0} k\,\hat{T}_{1\omega} = T_\delta(J_{0\omega},\omega)\cdot J_{1\omega} \tag{S12}$$

where $T_\delta(J_{0\omega},\omega)$ is a function that only depends on the DC bias $J_{0\omega}$, and ω . A full expression of $T_\delta(J_{0\omega},\omega)$ is shown in Equation (S14).

Although the second term on the right-hand side of Equation (S11) depends on applied current, our numerical calculations prove that the dependence is very weak, and it can be estimated as the temperature response with zero current (i.e. the conventional FDTR response). We numerically calculated the dependence of the amplitude of the second term on the right-hand side of Equation (S11) on $J_{0\omega}$ and $J_{1\omega}$ as shown in Fig. S1. The relative deviation caused by applied current on the second term's numerical value was found to be less than 0.7% of the value with zero current for the highly p-doped Si within the range of current density of our interest. Therefore, we can estimate the value of this integral as the surface temperature profile caused by the external periodic heat source without thermoelectric effects.

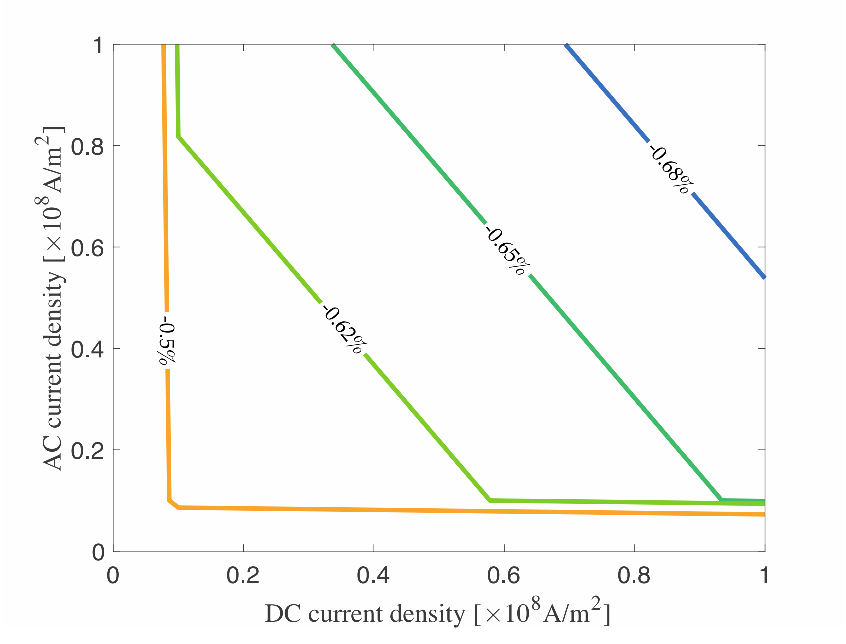

Figure S1. Relative amplitude deviation between the numerical value of the second term in right-hand side of Equation (S11) and the zero current first harmonic temperature caused by heat input. The results reveal that within the range of current density pertinent to our study, the applied current causes only a minimal variation of less than 0.7% in this term's value, so that it is safe for us to neglect it.

Temperature is measured in FDTR by a second coincident laser beam known as the probe laser. The probe laser returns a weighted average of the surface temperature, as given by

$$T = \int_{0}^{+\infty} w(r)\theta(r)dr \tag{S13}$$

where $w(r)$ is the radial weight function (gaussian for our probe laser).

Assuming the current uniformly distributes on the heated top surface, the measured first harmonic temperature response will be Equation 4, $T_{1\omega} = T_{\delta}(J_{0\omega}, \omega) \cdot J_{1\omega} + T_{1\omega 0}$, where

$$\begin{aligned}
T_{\delta}(J_{0\omega}, \omega) = {} & 2\, exp\left(z_1\sqrt{i\omega/\alpha_m}\right) G \Bigg(exp\Bigg(z_1 \\
& + z_2\sqrt{i\omega/\alpha_{Si}}\, \alpha_{Si} J_{0\omega} 4i\left(\kappa_{Si} + (z_2 - z_1) J_{0\omega} S\right)\Bigg) \rho\sqrt{i\omega/\alpha_{Si}} \\
& + exp\left(2z_2\sqrt{i\omega/\alpha_{Si}}\right) \Big(J_{0\omega}^2 S\rho \Big(-0.5 z_1^2\omega + z_1 z_2\omega - 0.5 z_2^2\omega + 2i\alpha_{Si} z_1\sqrt{i\omega/\alpha_{Si}} \\
& - 2i\alpha_{Si} z_2\sqrt{i\omega/\alpha_{Si}}\Big) + \kappa_{Si}\left(-ST_C\omega - 2i\alpha_{Si} J_{0\omega}\rho\sqrt{i\omega/\alpha_{Si}}\right)\Big) \\
& + exp\left(2z_1\sqrt{i\omega/\alpha_{Si}}\right) \Big(J_{0\omega}^2 S\rho \Big(0.5 z_1^2\omega - z_1 z_2\omega + 0.5 z_2^2\omega + 2i\alpha_{Si} z_1\sqrt{i\omega/\alpha_{Si}} \\
& - 2i\alpha_{Si} z_2\sqrt{i\omega/\alpha_{Si}}\Big) + \kappa_{Si}\left(ST_C\omega - 2i\alpha_{Si} J_{0\omega}\rho\sqrt{i\omega/\alpha_{Si}}\right)\Big)\Bigg) \\
& /(\kappa_{Si} - z_1 J_{0\omega} S \\
& + z_2 J_{0\omega} S)\omega \Bigg(exp\left(2z_1\sqrt{i\omega/\alpha_m} + 2z_1\sqrt{i\omega/\alpha_{Si}}\right) \Big(-\kappa_m\sqrt{i\omega/\alpha_m} \Big(J_{0\omega} S \\
& - k_{Si}\sqrt{i\omega/\alpha_{Si}}\Big) + G\left(-J_{0\omega} S - \kappa_m\sqrt{i\omega/\alpha_m} + \kappa_{Si}\sqrt{i\omega/\alpha_{Si}}\right)\Big) \\
& + exp\left(2z_2\sqrt{i\omega/\alpha_{Si}}\right) \Big(-\kappa_m\sqrt{i\omega/\alpha_m}\left(J_{0\omega} S + \kappa_{Si}\sqrt{i\omega/\alpha_{Si}}\right) \\
& + G\left(J_{0\omega} S - \kappa_m\sqrt{i\omega/\alpha_m} + \kappa_{Si}\sqrt{i\omega/\alpha_{Si}}\right)\Big) \\
& + exp\left(2z_1\sqrt{i\omega/\alpha_{Si}}\right) \Big(\kappa_m\sqrt{i\omega/\alpha_m}\left(J_{0\omega} S + \kappa_{Si}\sqrt{i\omega/\alpha_{Si}}\right) \\
& + G\left(-J_{0\omega} S + \kappa_m\sqrt{i\omega/\alpha_m} + \kappa_{Si}\sqrt{i\omega/\alpha_{Si}}\right)\Big) \\
& + exp\left(2z_1\sqrt{i\omega/\alpha_m} + 2z_2\sqrt{i\omega/\alpha_{Si}}\right) \Big(\kappa_m\sqrt{i\omega/\alpha_m}\left(J_{0\omega} S + \kappa_{Si}\sqrt{i\omega/\alpha_{Si}}\right) \\
& + G\left(J_{0\omega} S + \kappa_m\sqrt{i\omega/\alpha_m} + \kappa_{Si}\sqrt{i\omega/\alpha_{Si}}\right)\Big)\Bigg)
\end{aligned} \tag{S14}$$

Supplementary Note 2: Effect of DC Current on Transient Active Cooling Performance

Equation (S14) signifies that T_δ is a function of the DC current. To investigate this correlation, we conduct experiments wherein a DC current is superimposed onto the 10 kHz AC current utilized for transient temperature cancellation measurement. Additionally, we conduct a comparative analysis between the measured T_δ's amplitude and the theoretical predictions derived from Equation (S14) (as depicted in Fig. S2). It is worth noting that the theoretical values exhibit a turning point at around 120 mA. We hypothesize that this turning point is linked to the optimal DC current in steady-state Peltier cooling. When the DC current falls below this optimal current, an increase in the DC current leads to a reduction in $T_{0\omega}$, consequently diminishing the transient Peltier heat flux since transient Peltier flux is $S_{Si}(T_{0\omega}J_{1\omega} + T_{1\omega}J_{0\omega})$. This, in turn, results in a decline in the performance of transient active cooling. Conversely, when the DC current surpasses the optimal current, an increment in the DC current raises $T_{0\omega}$, thereby amplifying the effectiveness of transient active cooling. Owing to constraints related to the bonding wire in our sample and the capabilities of the arbitrary function generator, we encountered difficulties in applying a DC offset at the anticipated turning point position. Nonetheless, our experimental results confirmed the trend of T_δ within the experimentally accessible range of DC current. Consequently, we find that when applying transient active cooling with limited DC supply, there is a trade-off between the transient active cooling capability and steady-state temperature. It is possible to elevate transient active cooling performance by introducing a negative DC offset onto the AC current; however, this concurrently results in an increase in the steady-state temperature. Conversely, applying a positive DC offset can lead to a reduction in steady-state temperature but concomitantly diminishes the efficacy of active cooling.

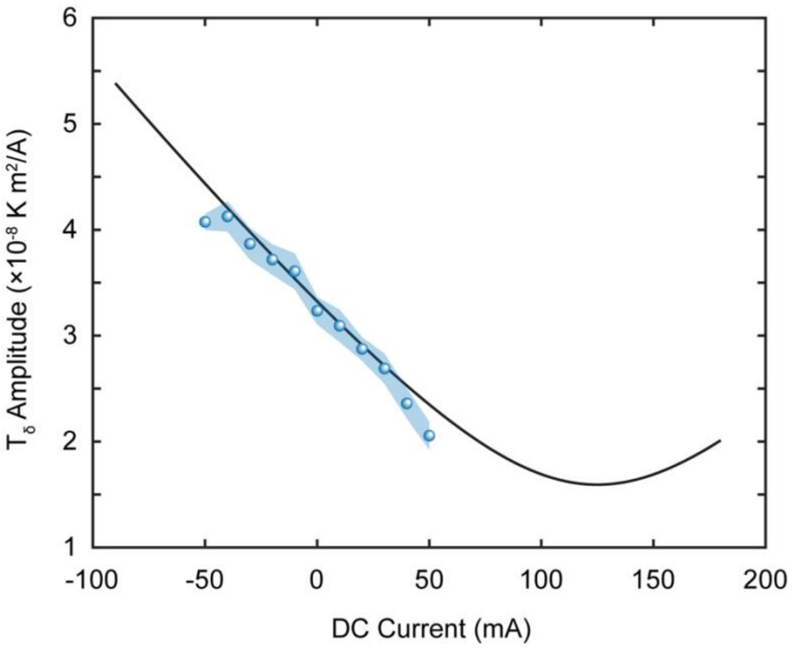

Figure S2. Dependence of T_δ's amplitude on DC offset at 10 kHz. Measured value (points) aligns with theoretical prediction (solid line) to the left of turning point (~ 120 mA).

Supplementary Note 3: Reduction of Thermal Boundary Conductance at Ni/p-Si Interface After Annealing

We investigate the influence of annealing on the thermal boundary conductance at the interface of Ni and p-Si, as examined through the analysis of phase-frequency data acquired via FDTR measurements. The outcomes of fitting FDTR data for individual samples (Fig, S3) show that the formation of Ni silicide formed during the RTA process at 600 °C will significantly reduce the thermal boundary conductance, decreasing from 382 ± 20 MW m^{-2} K^{-1} to 60 ± 17 MW m^{-2} K^{-1}.

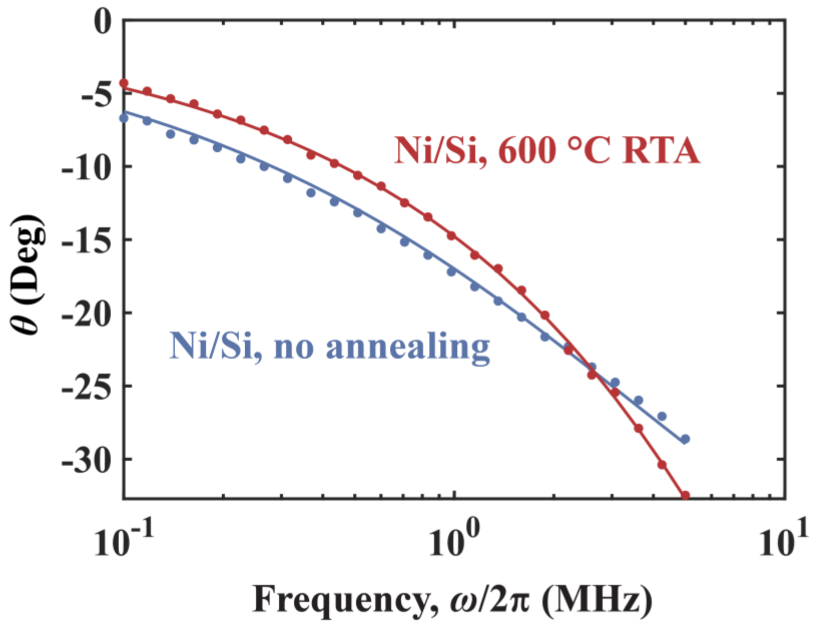

Figure S3. Comparison of FDTR data between annealed (red) and unannealed (blue) Ni/Si samples

Supplementary Note 4: Measurement of T_δ's Phase and Phase Distortion Resulting from Device's Parasitic Inductance

To determine dependence of T_δ's phase on frequency, we conduct linear regressions involving $\Delta T_{1\omega}$'s phase and $J_{1\omega}$'s phase at various frequencies, ranging from 100 kHz to 5 MHz (Fig. S4a). Notably, the slopes of these regressions for all frequencies closely approximate 1, aligning with Equation (4) as $\Delta T_{1\omega} = T_\delta(J_{0\omega}, \omega)J_{1\omega}$ and this relationship implies that $\phi(\Delta T_{1\omega}) = \phi(J_{1\omega}) + \phi(T_\delta(J_{0\omega}, \omega))$. The intercept values obtained from these regressions represent the phase value of T_δ for different frequency. We find the presence of parasitic inductance within the device package introduces an increasing phase delay as the frequency rises. Hence the phase distortion on T_δ resulting from the parasitic inductance is arctan($2\pi f$ L/R), where L is the value of inductance and R is device's resistance. This parasitic inductance is attributed to the Si TED's low resistance, compounded by the parasitic inductance stemming from bonding wires and Au/Ni contacts, and wires' geometric design. At high frequencies, this parasitic inductance gives rise to a substantial phase delay. The presence of this parasitic inductance will cause a significant discrepancy in the phase that we need to add on the AC current for active cancellation in experiments compared to theory. This discrepancy also contributes to the difference between measured data and analysis result in Fig. 2d. It is possible to easily determine the inductance value by high frequency electrical measurements that determine the correction to the phase at all frequencies accordingly. For our devices we fit the ratio of parasitic inductance to device resistance, L/R, as 0.25 $\mu\text{H}/\Omega$ as shown in Fig. S4b.

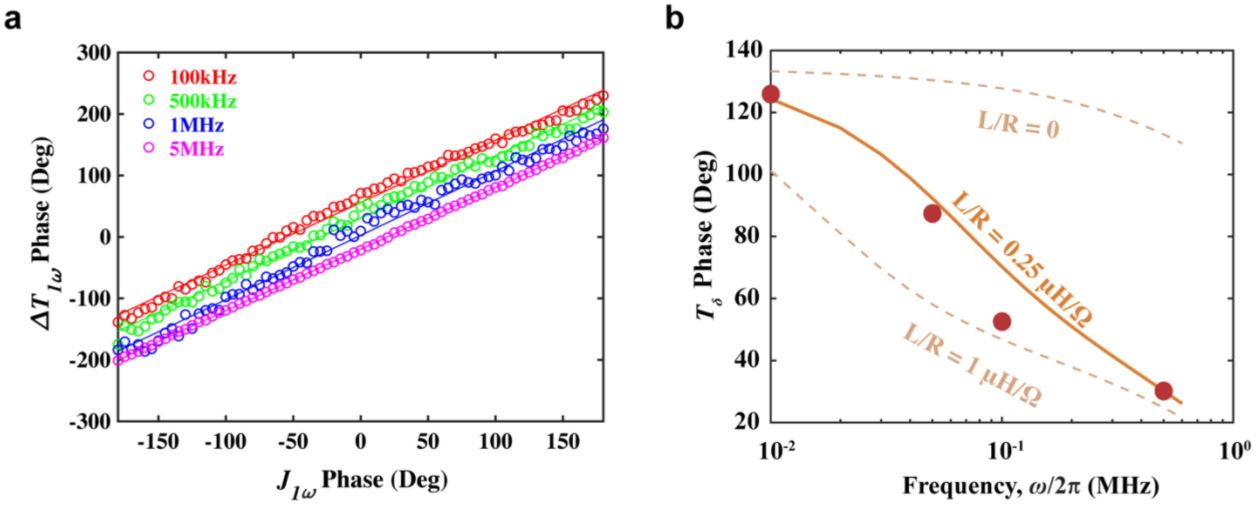

Figure S4. Measurement of T_δ's phase and its distortion resulting from parasitic inductance. **a** Measured dependence of $\Delta T_{1\omega}$'s phase on AC input's phase at different frequencies. **b** Dependence of T_δ's phase on heat flux's frequency. Solid line is numerical phase value assuming ratio between package parasitic inductance and device resistance is 0.25 μH/Ω. Dashed lines are numerical phase value for L/R= 0 and 1 μH/Ω respectively, for comparison purpose.

Supplementary Note 5: Derivation of COP_{trans} at High Frequency

To evaluate the temporal average power density of the first harmonic component of heat flux in the gaussian distribution, we estimate its peak heat flux as

$$q''_{\mathrm{peak}} = \frac{1}{\pi w_0^2} P_{\mathrm{laser}} \quad \text{(S15)}$$

so that for a sinusoidal heat flux its RMS value is

$$q''_{\mathrm{RMS}} = \frac{q''_{\mathrm{peak}}}{\sqrt{2}} = \frac{1}{\sqrt{2}\pi w_0^2} P_{\mathrm{laser}} \quad \text{(S16)}$$

When $T_{1\omega}$ is totally cancelled, the area average of the RMS of the electrical power applied on TED is

$$pTED_{\mathrm{RMS}} = \frac{I_{\mathrm{peak}}^2 R}{2A_{\mathrm{contact}}} \quad \text{(S17)}$$

where I_{peak} is the peak amplitude of AC current applied, R is the resistance of TED and A_{contact} is the hot end contact area. Equation (S17) can also be rewritten as

$$pTED_{\mathrm{RMS}} = \frac{J_{1\omega}^2 \rho_{Si} h_{\mathrm{TED}}}{2} \quad \text{(S18)}$$

where h_{TED} is the height of the TED.

The ratio of q''_{RMS} to $pTED_{\mathrm{RMS}}$ determines the coefficient of performance of device's transient active cooling (COP_{trans}). We can derive the expression of COP_{trans} from Equation (4), (S15), (S16) and (S18)

$$\mathrm{COP}_{\mathrm{trans}} = \frac{\sqrt{2}|T_\delta(J_{0\omega}, \omega)|^2 q''_{\mathrm{peak}}}{|T_{1\omega 0}|^2 \rho_{Si} h_{\mathrm{TED}}} \quad \text{(S19)}$$

Note that at high frequency ($f \gg \frac{\alpha_{\mathrm{TE}}}{2\pi z_2^2}$, which is ~13.2 kHz for our sample), $|T_\delta(J_{0\omega}, \omega)|$ is approximately proportional to $\exp\left(-z_1\sqrt{\omega/\alpha_m}\right)/\sqrt{\omega}$ based on Eqn. (S14) and $T_{1\omega 0} \propto q''_{\mathrm{peak}}/\sqrt{\omega}$ (Equation (13) in Ref. 15), so that

$$\mathrm{COP_{trans}} \propto \frac{\exp\left(-2z_1\sqrt{\frac{2\pi f}{\alpha_m}}\right)}{q''_{\mathrm{peak}}} \qquad (S20)$$

Hence $\mathrm{COP_{trans}}$ decrease exponentially with increasing $\sqrt{f}$.

Supplementary Note 6: Comparison of COP_{trans} between p-Si and p-Bi_2Te_3 active cooling devices

Values of p type Bi_2Te_3 we used to calculate p-Bi_2Te_3 active cooling devices' COP_{trans} are listed in Table S3.

S (μV/K)	κ (W/(m·K))	ρ (μΩ·m)	α ($\times10^{-8}$ m^2/s)	G (MW/(m^2·K))
174[1]	1.84[1]	17.8[1]	168[2]	1[3]

Table S3. Values of p-Bi_2Te_3's properties for its active cooling devices' COP_{trans} calculation

Figure S5a compares the COP_{trans} of active cooling devices made of p-Si and p-Bi_2Te_3. The COPtrans of p-Si devices surpasses that of p-Bi_2Te_3 devices due to its significantly higher thermal conductivity. Additionally, as the thickness of the hot-side metal electrode increases, the COP_{trans} of both materials rises since the thicker metal electrode enhances the devices' passive cooling performance. However, increasing the metal thickness also diminishes the device's transient temperature modulation capability as quantified by the amplitude of T_δ shown in Fig. S5b. This decline occurs because periodic Peltier cooling, generated at the interface between the thermoelectric materials and the metal contact, induces an exponentially decaying temperature profile in the metal layer[4]. Consequently, the thicker the metal electrode, the lower the temperature modulation capability the devices will exhibit on the surface.

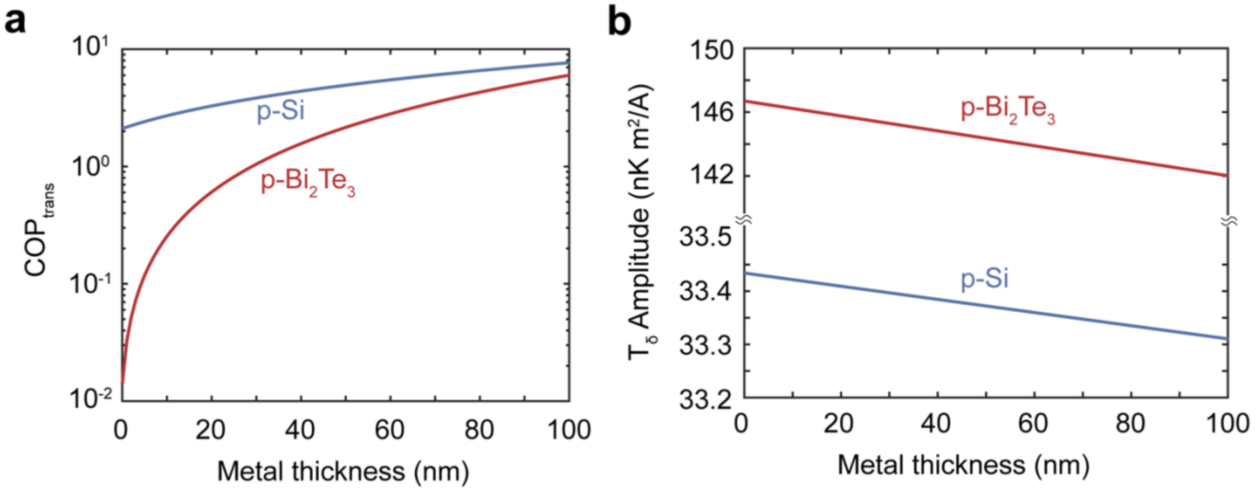

Figure S5. Effect of hot-side metal electrode's thickness on COP_{trans}. a COP_{trans} comparison between p-Si and p-Bi_2Te_3 active cooling devices at frequency of 10 kHz when the RMS value of the external transient heat source's heat flux amplitude is 10 kW/cm^2. **b** T_δ decreases as hot-side metal electrode thickness increases at frequency of 10 kHz.

Supplementary Note 7: Transient Temperature Cancellation at High Frequency (> 100 kHz)

Figure S6 illustrates the transient temperature cancellation observed at higher operating frequencies. As the operating frequency increases, the heat flux that can be cancelled is reduced for the same AC amplitude. This phenomenon stems from the COP_{trans} decreasing with the increase in frequency at high frequency.

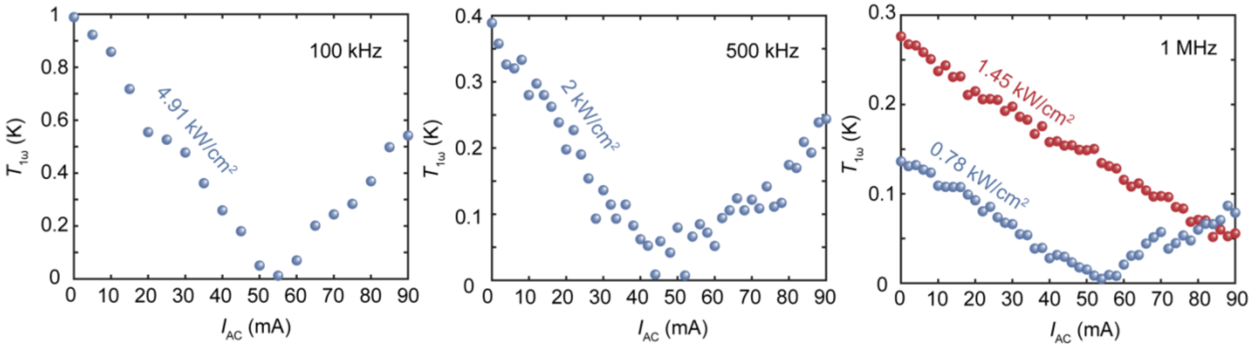

Figure S6. Transient temperature cancellation observed under higher frequency (>100 kHz)

Supplementary Note 8: Discussion on the Simplification of Neglecting Higher Order Transient Temperature Components in Model Study

In the model study, we neglect higher order harmonic temperature and its influence on thermoelectric effect to obtain a closed-form result for the 1st harmonic temperature response. In reality the 2nd harmonic from Joule heating will generate a 2nd harmonic temperature response, $T_{2\omega}$, that grows with applied current. This $T_{2\omega}$ will also affect $T_{1\omega}$ through the thermoelectric heat flux $SJ_{1\omega}T_{2\omega}$. Nevertheless, Fig. 3b and 3c show that the transient temperature modulation capability, T_{δ}, is independent of the amplitude of the AC current. In Fig. 3b's upper panel, the linear reduction of $T_{1\omega}$ indicates that T_{δ}'s amplitude is independent AC current. In Fig. 3c, the linear trajectory of $T_{1\omega}$ in polar coordinates indicates that the phase of T_{δ} has not changed. Therefore, data shown in Fig. 3 indicate that higher order harmonic temperatures are small and do not significantly inhibit the first harmonic temperature active cooling process.

Reference

1 Rowe, D. M. *CRC handbook of thermoelectrics*. (CRC press, 2018).

2 Arisaka, T., Otsuka, M. & Hasegawa, Y. Measurement of thermal conductivity and specific heat by impedance spectroscopy of Bi2Te3 thermoelectric element. *Review of Scientific Instruments* **90** (2019).

3 Högblom, O. & Andersson, R. Analysis of thermoelectric generator performance by use of simulations and experiments. *Journal of Electronic Materials* **43**, 2247-2254 (2014).

4 Regner, K. T., Majumdar, S. & Malen, J. A. Instrumentation of broadband frequency domain thermoreflectance for measuring thermal conductivity accumulation functions. *Review of Scientific Instruments* **84** (2013). https://doi.org:10.1063/1.4808055
